# Identification of miRNA-103 in the Cellular Fraction of Human Peripheral Blood as a Potential Biomarker for Malignant Mesothelioma – A Pilot Study

**DOI:** 10.1371/journal.pone.0030221

**Published:** 2012-01-11

**Authors:** Daniel G. Weber, Georg Johnen, Oleksandr Bryk, Karl-Heinz Jöckel, Thomas Brüning

**Affiliations:** 1 Center of Molecular Medicine, Institute of Prevention and Occupational Medicine of the German Social Accident Insurance - Institute of the Ruhr-Universität Bochum (IPA), Bochum, Germany; 2 Institute of Medical Informatics, Biometry and Epidemiology, University Duisburg-Essen, Essen, Germany; Virginia Commonwealth University, United States of America

## Abstract

**Background:**

To date, no biomarkers with reasonable sensitivity and specificity for the early detection of malignant mesothelioma have been described. The use of microRNAs (miRNAs) as minimally-invasive biomarkers has opened new opportunities for the diagnosis of cancer, primarily because they exhibit tumor-specific expression profiles and have been commonly observed in blood of both cancer patients and healthy controls. The aim of this pilot study was to identify miRNAs in the cellular fraction of human peripheral blood as potential novel biomarkers for the detection of malignant mesothelioma.

**Methodology/Principal Findings:**

Using oligonucleotide microarrays for biomarker identification the miRNA levels in the cellular fraction of human peripheral blood of mesothelioma patients and asbestos-exposed controls were analyzed. Using a threefold expression change in combination with a significance level of p<0.05, miR-103 was identified as a potential biomarker for malignant mesothelioma. Quantitative real-time PCR (qRT-PCR) was used for validation of miR-103 in 23 malignant mesothelioma patients, 17 asbestos-exposed controls, and 25 controls from the general population. For discrimination of mesothelioma patients from asbestos-exposed controls a sensitivity of 83% and a specificity of 71% were calculated, and for discrimination of mesothelioma patients from the general population a sensitivity of 78% and a specificity of 76%.

**Conclusions/Significance:**

The results of this pilot study show that miR-103 is characterized by a promising sensitivity and specificity and might be a potential minimally-invasive biomarker for the diagnosis of mesothelioma. In addition, our results support the concept of using the cellular fraction of human blood for biomarker discovery. However, for early detection of malignant mesothelioma the feasibility of miR-103 alone or in combination with other biomarkers needs to be analyzed in a prospective study.

## Introduction

Malignant mesothelioma (MM) is an incurable cancer of the serous membranes and is highly associated with asbestos exposure. In the United States, 18,068 MM cases were reported between 1999 and 2005 [1], with a projection of approximately 71,000 MM cases by 2054 [2]. Similar trends were also predicted for Western Europe, with 250,000 deaths in the next 30–35 years [3], and in many other countries worldwide [4]. The latency period from asbestos exposure to tumor development is up to 40 years and symptoms usually appear in late stages of the disease. Early diagnosis of tumors generally leads to more effective therapies. Therefore, the same might be true for MM [5]. Biomarkers have the potential to facilitate an early diagnosis of cancer. However, proper biomarkers need to be sensitive enough to detect early stages of the tumors and highly specific to avoid false-positive results in cancer-free individuals. Unfortunately, none of the currently examined biomarkers, such as SMRP (soluble mesothelin-related peptides), CA 125, and CYFRA 21-1, either alone or in combination, serve as predictors for the early diagnosis of MM [6], [7].

The discovery of microRNAs (miRNAs) provided new opportunities for the use of biomarkers in the diagnosis of cancer [8]. MiRNAs are small (∼22 nt) noncoding RNA molecules playing a central role in the regulation of gene expression [9]. In cancer, miRNAs act as either oncogenes or tumor suppressors [10]. Altered miRNA expression has been reported in several human malignancies and differences in expression between tumor tissues and their benign counterparts could be useful for cancer diagnostics [11], [12]. Several analyses of miRNA expression in MM have already been performed resulting in the identification of miRNAs as potential biomarkers [13]–[18]. However, most of the studies analyzed miRNAs in tissues, while proper biomarkers should be detectable in easily accessible samples. Alternatively, miRNAs have also been found in body fluids [19]–[22]. Human blood in particular is the preferred source of biomarkers due to the minimally-invasive nature of sample collection and the vascularization of most tissues, including tumors [23]. In human peripheral blood, miRNAs were not only detectable in serum or plasma, but also in the cellular fraction [24]. Recently, Häusler et al. showed that neoplastic diseases generate characteristic miRNA fingerprints in the cellular fraction of human peripheral blood [25].

In this pilot study, we analyzed miRNA expression in the cellular fraction of peripheral human blood of malignant mesothelioma patients (MMP) and asbestos-exposed controls (AEC) using oligonucleotide microarrays. Significantly altered miRNAs were selected as potential blood-based biomarkers for MM and evaluated by quantitative real time-PCR (qRT-PCR) in MMP, AEC, and additionally controls from the general population (CGP).

## Methods

### Ethics statement

All participants provided written informed consent. The study was designed according to rules guarding patient privacy and with the approval from the ethics committee of the Ruhr-Universität Bochum (reference number 3217-08).

### Study population

The study group consisted of 23 patients with diagnosed pleural MM (mean age 66 years, range 34–84 years). Patients were not treated by surgery, chemotherapy, or radiation therapy before sample collection. The histological subtypes were: one sarcomatoid, seven biphasic, and twelve epithelioid mesotheliomas. In three cases, the histological subtype was unknown. Detailed characteristics of patients with diagnosed mesothelioma are listed in Table 1. The cancer-free control groups consisted of 17 subjects formerly exposed to asbestos (mean age 68 years, range 47–80 years) and 25 volunteers from the general population (mean age 70 years, range 56–84 years). The volunteers were matched to the mesothelioma group by age, gender, and smoking status (smoker, ex-smoker, non-smoker). Detailed characteristics of the controls are summarized in Table 2 and 3.

**Table 1:** Characteristics of malignant mesothelioma patients (MMP).

Sample	Age	Sex	Smoking status	Histological subtype*
MMP000	56	Female	Ex	Epithelioid
MMP001	84	Male	Ex	Biphasic
MMP002	54	Male	Ex	Biphasic
MMP003	68	Male	No	n.a.
MMP004	66	Female	No	Biphasic
MMP005	83	Female	No	Biphasic
MMP011	72	Male	Ex	Biphasic
MMP012	73	Male	Ex	n.a.
MMP013	70	Male	No	n.a.
MMP014	68	Male	No	Epithelioid
MMP026	76	Female	Ex	Epithelioid
MMP027	34	Male	Ex	Epithelioid
MMP028	70	Male	No	Biphasic
MMP029	59	Male	No	Epithelioid
MMP039	69	Female	No	Epithelioid
MMP040	74	Male	No	Epithelioid
MMP041	73	Male	No	Epithelioid
MMP042	72	Male	No	Sarcomatoid
MMP044	66	Male	Ex	Epithelioid
MMP045	73	Male	Ex	Biphasic
MMP056	53	Male	No	Epithelioid
MMP057	56	Male	Ex	Epithelioid
MMP060	51	Male	n.a.	Epithelioid

*All tumors are of pleural origin, n.a.: not available.

**Table 2:** Characteristics of asbestos-exposed controls (AEC).

Sample	Age	Sex	Smoking status
AEC001	61	Male	Yes
AEC002	69	Male	Yes
AEC004	67	Male	Ex
AEC005	68	Male	No
AEC008	77	Male	Yes
AEC010	76	Male	No
AEC011	60	Male	Ex
AEC012	76	Male	No
AEC014	79	Male	No
AEC015	69	Male	Ex
AEC016	67	Male	Yes
AEC017	56	Male	Ex
AEC018	80	Male	No
AEC019	67	Male	Yes
AEC020	47	Female	No
AEC021	74	Male	Ex
AEC022	62	Male	Yes

**Table 3:** Characteristics of controls from the general population (CGP).

Sample	Age	Sex	Smoking status
CGP001	63	Female	No
CGP002	60	Male	Yes
CGP003	57	Male	Ex
CGP004	70	Male	Ex
CGP005	64	Female	Yes
CGP006	59	Female	Yes
CGP007	77	Male	No
CGP008	72	Male	Ex
CGP009	64	Male	Ex
CGP010	72	Male	Yes
CGP011	71	Female	No
CGP012	58	Male	Yes
CGP013	76	Male	Ex
CGP014	75	Male	Ex
CGP015	66	Female	Ex
CGP016	84	Male	No
CGP017	77	Male	Ex
CGP018	56	Male	Ex
CGP019	62	Male	Ex
CGP020	77	Male	Ex
CGP021	81	Male	Ex
CGP022	77	Male	No
CGP023	71	Male	Ex
CGP024	83	Female	Ex
CGP025	66	Female	Ex

According to Wang et al., the case and control samples were not split into training and test sets [8].

### RNA isolation

From each participant, peripheral blood was collected in 9.0 ml S-Monovette EDTA gel tubes (Sarstedt, Nümbrecht, Germany). Within 30 minutes after blood collection, tubes were centrifuged at 2000×g for 10 minutes at room temperature. The cellular fraction was separated from plasma and immediately stored at −20°C until RNA isolation. Samples were thawed at room temperature and RNA isolation from 0.5 ml of the cellular fraction was performed using the RiboPure-Blood Kit according to the Alternate protocol: Isolation of Small RNAs (Applied Biosystems, Foster City, CA, USA).

Concentration of isolated RNA was quantified by measuring the absorbance at 260 nm using a NanoDrop ND-100 spectrophotometer (Thermo Scientific, Waltham, MA, USA).

### Oligonucleotide microarrays

Oligonucleotide microarrays were purchased from the Norwegian Microarray Consortium (www.microarray.no). The microarrays were spotted using the mirVana miRNA Probe Set v2.0 (Ambion, Austin, TX, USA), which is based on the miRBase Sequence Database v8.0 [26]. Probes were spotted in triplicates using Pronto! Microarray spotting solution (Corning, Corning, NY, USA) on CMT UltraGAPS (Corning) microarray slides. Hybridization of the microarrays was performed using the microarray hybridization station HS 400 Pro (Tecan, Männedorf, Switzerland) according to Liu et al. [27]. Microarray scanning at 635 nm and signal definition were performed as described previously [28].

Labeling of 1 µg RNA was performed using 5′-phosphate-cytidyl-cytidyl-Cy5-3′ (Eurogentec, Cologne, Germany) according to the miRNA Microarray System Protocol (Agilent Technologies, Santa Clara, CA, USA). Labeled RNA was purified using a mini Quick Spin Column (Roche, Grenzach-Wyhlen, Germany) centrifuging at 1000×g for 10 minutes at room temperature.

Microarray platform and experimental data were deposited in the public database Gene Expression Omnibus (GEO) (https://www.ncbi.nlm.nih.gov/geo/). The series accession number is GSE29707.

### Microarray data analysis

Generated data were analyzed using the software GeneSpring GX 11.0 (Agilent Technologies). In brief, data transformation measurements less than 0.01 were set to 0.01 and normalization per microarray was performed using the 50th percentile. For analysis of altered miRNA expression between MMP and AEC a fold change of 3.0 was used as threshold. Altered miRNAs with fold-changes ≥3.0 were used for hierarchical clustering using Euclidian distance and Ward's linkage as parameters. Statistical differences were analyzed utilizing the Mann-Whitney unpaired test. For p-value correction the Westfall-Young-Permutation method was used.

The stability of all human miRNAs throughout all samples was analyzed using NormFinder [29]. According to Peltier and Latham modified Z-scores were used as input data for NormFinder analysis [30].

### Quantitative real time-PCR (qRT-PCR)

TaqMan miRNA Assays (Applied Biosystems) were used for quantitative miRNA expression analysis on a 7900 HT Fast Real-Time PCR System (Applied Biosystems) as described previously [23], [31]. 10 ng RNA and 5 µl cDNA were used as templates for the RT reaction and PCR reaction, respectively. Samples were analyzed in duplicate and non-template controls were included in all assays.

A fixed threshold of 0.2 was used for cycle threshold (Ct) estimation [32]. Ct values ≥35 were considered to be under the detection limit [33] and marked as 35 for calculation [34]. Ratios were calculated for normalization [23] and the expression value was expressed as 2^−Ratio^ equivalent to 2^−dCt^ [35]. Mann-Whitney unpaired tests were performed to examine differences in relative miRNA expression of MMP vs. AEC, MMP vs. CGP, and AEC vs. CGP as well as for subgroup comparisons regarding gender, smoking status, and histological subtypes. The association of age and miRNA levels was assessed using the Spearman correlation coefficient. Statistical analyses were performed using Prism 5 (GraphPad Software Inc., San Diego, CA, USA).

## Results

### Deregulated miRNAs in the cellular fraction of peripheral blood of MM patients

**Figure 1:**
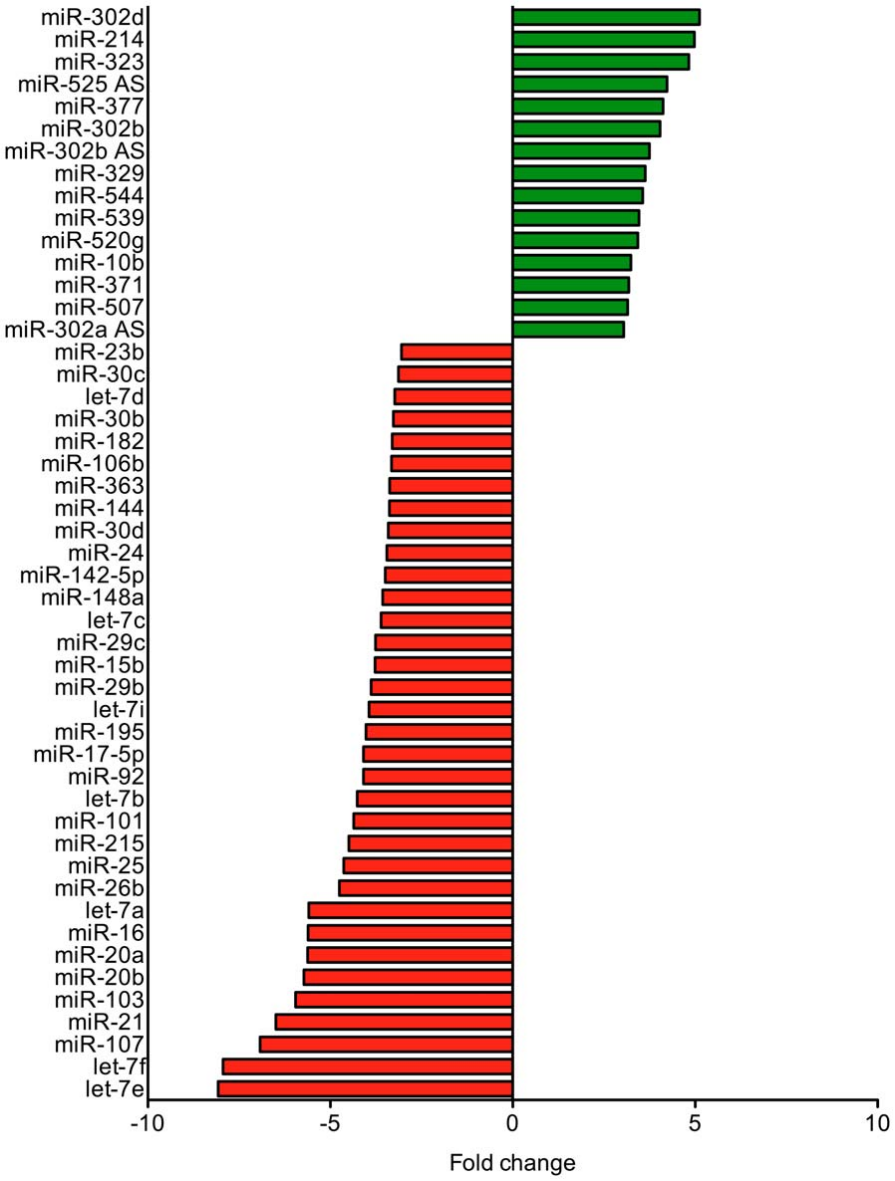
Deregulated miRNAs in the cellular fraction of human blood of mesothelioma patients and asbestos-exposed controls. Differences in miRNA expression were at least threefold. In patients with diagnosed malignant mesothelioma 15 miRNAs were up-regulated (green) and 34 miRNAs were down-regulated (red).

**Figure 2:**
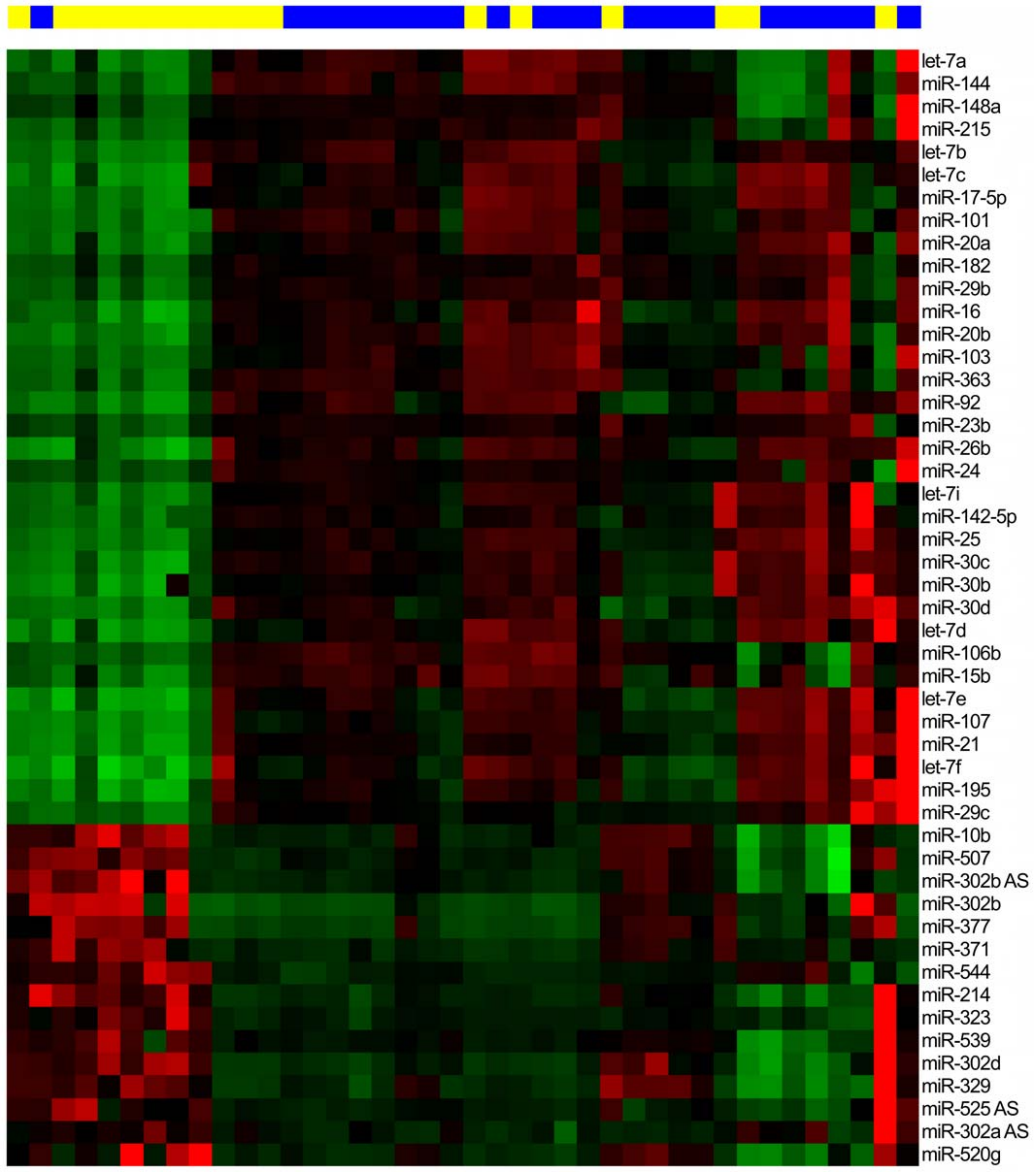
Heat map of miRNA expression of malignant mesothelioma patients and asbestos-exposed controls. Cluster analysis was performed using miRNA expressions with more than a threefold change in the cellular fraction of human peripheral blood samples. Samples of malignant mesothelioma patients were marked blue and samples of asbestos-exposed controls yellow.

The expression profiles of 328 human miRNAs were determined in 23 MMP and 17 AEC using oligonucleotide microarrays. In MMP, 49 miRNAs were deregulated with more than a threefold change in comparison to AEC. In particular, 34 miRNAs were down-regulated and 15 miRNAs were up-regulated. The deregulated miRNAs and the corresponding fold changes are presented in Figure 1. The 49 miRNAs with more than threefold change were used for hierarchical cluster analysis utilizing Euclidian distance and Ward's linkage (Figure 2).

Utilizing the Mann-Whitney unpaired test for statistical significance and the Westfall-Young-Permutation method for p-value correction, all deregulated miRNAs with more than a threefold change were analyzed. Only two of 49 miRNAs, miR-20a (p = 0.0101) and miR-103 (p = 0.0303), showed a significant down-regulation in MM.

### miR-20a and miR-103 as potential biomarkers for MM

The expression profiles of 328 human miRNAs in 23 MMP and 17 AEC samples were analyzed to reveal the most stable miRNA as reference. Using NormFinder, miR-125a was identified as the most stable miRNA in the analyzed set of MMP and AEC samples. Thus, miR-125a was used as the reference to normalize the raw Ct values of miR-20a and miR-103 obtained in the qRT-PCR analysis.

**Figure 3:**
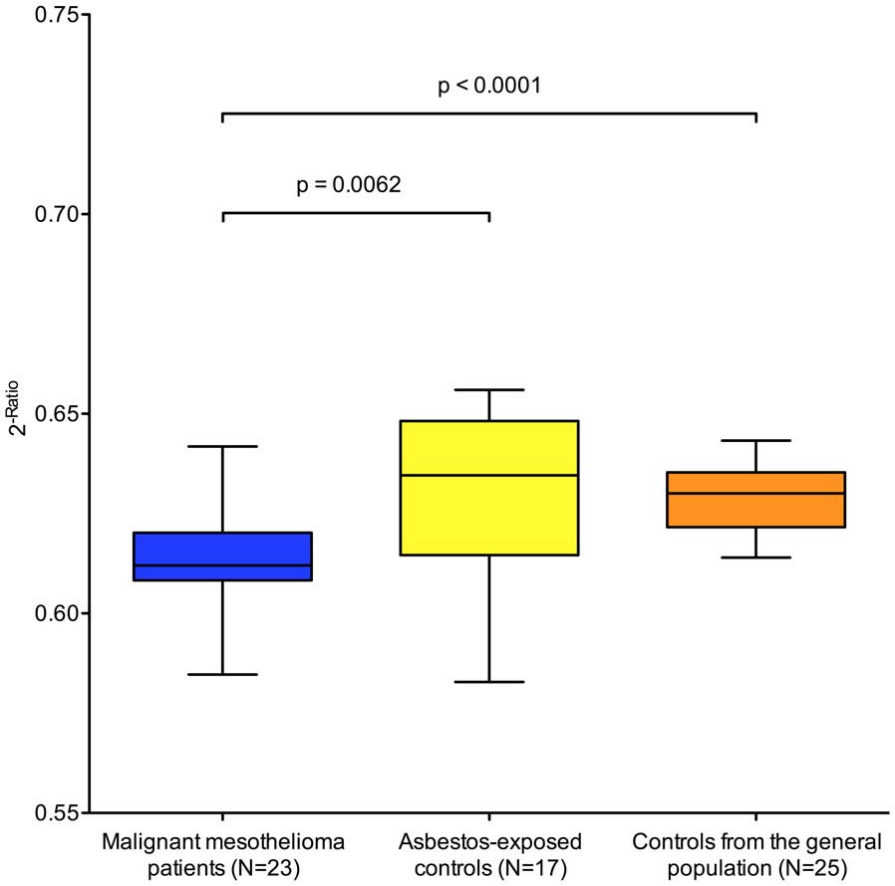
Box plots of relative expression of miR-103. Relative expression of miR-103 in the cellular fraction of human peripheral blood of malignant mesothelioma patients (blue), asbestos-exposed controls (yellow), and controls from the general population (orange). Expression values were normalized to miR-125a and expressed as 2^−Ratio^. Mann-Whitney test was performed to examine group differences.

Using normalized Ct values of miR-20a the median value and interquartile range (IQR) were 0.668 (IQR 0.659–0.676) for MMP and 0.682 (IQR 0.657–0.695) for AEC, but the observed difference was not significant (data not shown). Median value of miR-103 for MMP was 0.612 (IQR 0.608–0.620) and for AEC 0.635 (IQR 0.615–0.648) and the difference was significant (p = 0.0062). For CGP median value of miR-103 was 0.630 (IQR 0.622–0.635) and differences were significant for MMP vs. CGP (p<0.0001) but not for AEC vs. CGP (Figure 3).

**Figure 4:**
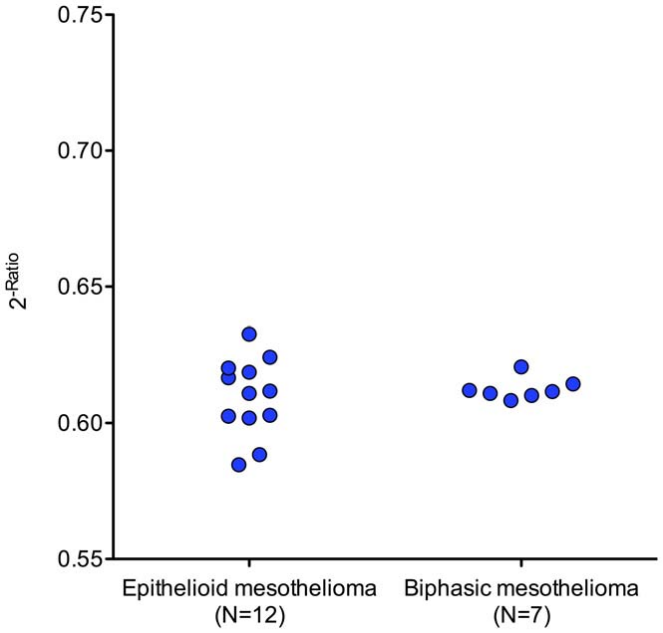
Scatter plot of relative expression of miR-103 in histological subtypes of malignant mesothelioma. Relative expression of miR-103 in the cellular fraction of human peripheral blood of patients with epithelioid and biphasic mesothelioma. One sarcomatoid mesothelioma case and three cases without available histological subtype were excluded from analysis. Expression values were normalized to miR-125a and expressed as 2^−Ratio^.

Using normalized Ct values of miR-103, differences between histological subtypes were analyzed. Median value of miR-103 was 0.611 (IQR 0.602–0.620) for epithelioid mesothelioma and 0.612 (IQR 0.610–0.614) for biphasic mesothelioma (Figure 4). No significant differences between both subtypes could be observed. The sarcomatoid subtype comprises only one sample and was not included in the analysis.

**Figure 5:**
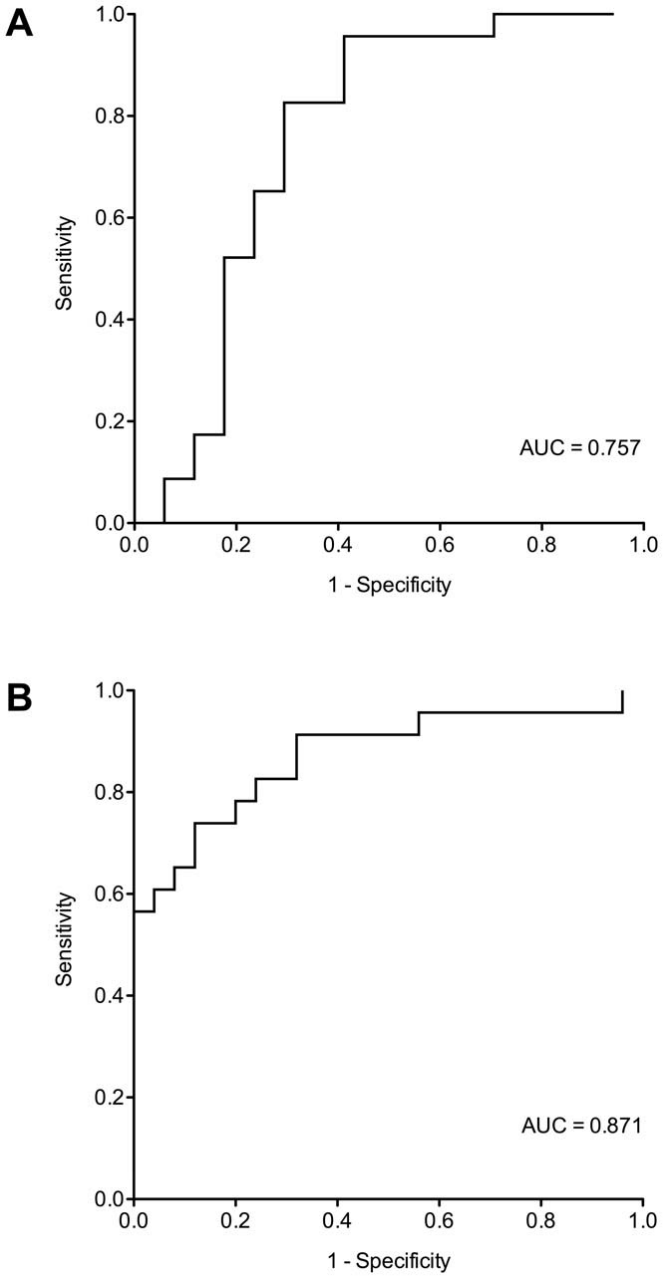
Receiver operating characteristics (ROC) curves of miR-103. The area under curve (AUC) was determined for miR-103 in the cellular fraction of human peripheral blood of (A) malignant mesothelioma patients and asbestos-exposed controls and (B) malignant mesothelioma patients and controls from the general population.

Using ROC analysis for miR-103, an AUC value of 0.757 (95% CI 0.586–0.929, p = 0.0060) could be calculated for MMP vs. AEC (Figure 5 A) and an AUC of 0.871 (95%CI 0.766–0.977, p<0.0001) for MMP vs. CGP (Figure 5 B). A proper cut-off for miR-103 using a 2^−Ratio^ value of 0.621 was determined to discriminate MMP from AEC revealing a sensitivity of 83% and specificity of 71%. Using the cut-off to discriminate MMP from CGP reveals a sensitivity of 78% and a specificity of 76%.

**Figure 6:**
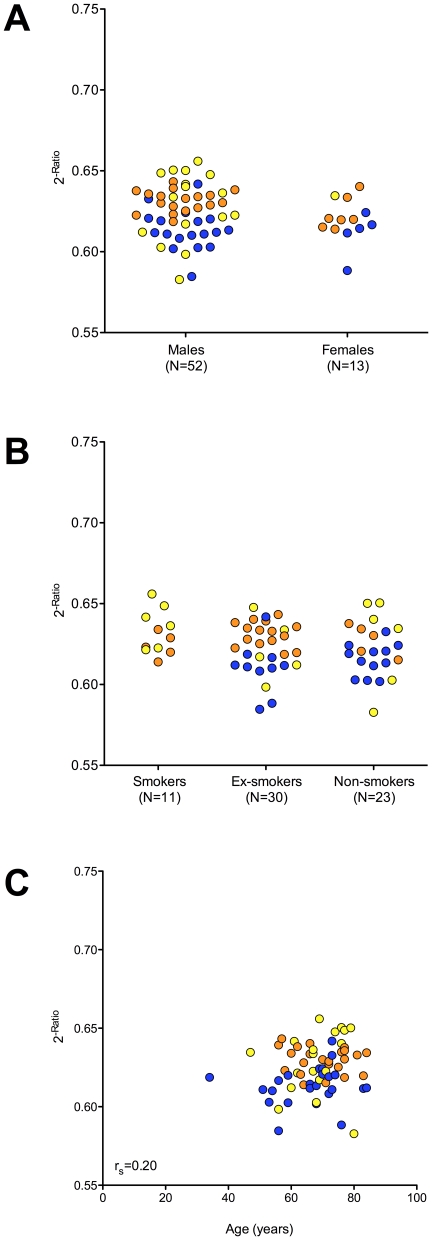
Scatter plots of relative expression of miR-103. Relative expression of miR-103 in the cellular fraction of human peripheral blood of malignant mesothelioma patients (blue), asbestos-exposed controls (yellow), and controls from the general population (orange) regarding (A) gender, (B) smoking status, and (C) age. The smoking status of one MMP case was not available. Expression values were normalized to miR-125a and expressed as 2^−Ratio^. Mann-Whitney tests were performed to examine differences between groups. Spearman correlation coefficient was calculated to evaluate association between miR-103 levels and age.

Subgroup comparisons regarding gender, smoking status, and age were performed using normalized Ct values of miR-103. For male subjects the median value of miR-103 was 0.625 (IQR 0.612–0.636) and for female subjects 0.620 (IQR 0.614–0.629) (Figure 6 A). Regarding the smoking status the median value of miR-103 was 0.629 (IQR 0.622–0.642) for smokers, 0.624 (IQR 0.612–0.635) for ex-smokers, and 0.621 (IQR 0.612–0.634) for non-smokers, (Figure 6 B). Using the Mann-Whitney test, no significant differences could be observed for gender and smoking status. The Spearman correlation coefficient was used to reveal potential correlation between age and miRNA levels, revealing a marginal association between age and miRNA level (r_s_ = 0.20, 95% CI −0.05–0.43), (Figure 6 C).

## Discussion

Mesothelioma is a fatal cancer and chiefly associated with former asbestos exposure. The tumor is commonly detected in late stages of the disease. Blood-based biomarkers would greatly improve diagnosis and early detection of MM [36]. It has been suggested that miRNAs are promising biomarkers for several human malignancies [24], [37] and the same might be true for MM. Most studies that analyzed miRNA expression in MM used tissues. However, appropriate biomarkers need to be detectable in easily accessible body fluids like peripheral blood. To date, miRNAs as blood-based biomarkers for diagnosis of MM have not been extensively investigated. In a most recent study, Santarelli et al. analyzed miR-126 as a free circulating nucleic acid in serum [15].

In this study, we analyzed the miRNA expression of MMP and AEC in the cellular fraction of human peripheral blood to identify specific miRNAs as potential biomarkers for MM. To our knowledge, this is the first study analyzing miRNA in the cellular fraction of human blood for biomarker evaluation of MM. Our experimental design followed the recent study of Häusler et al. who utilized this concept for miRNA expression analysis in ovarian cancer [25]. They assumed that cancer-induced miRNA profiles in cellular blood cells might already be detectable at early stages in the development of tumors [25], because it was shown that the formation of a pre-metastatic niche by hematopoietic cells is an early event of tumorigenesis and metastasis [38]. Häusler et al. assumed that free circulating tumor-specific miRNAs in plasma or serum may be partly masked by high amounts of cellular miRNAs, but this loss of information is compensated for by the information revealed from the cellular fraction [25]. For ovarian cancer they believe, that stromal and myeloid progenitors or regulatory T cells, which are recruited to the tumor site, may significantly contribute to the miRNA profiles [25] and the same might be true for MM. Further, tumors can send immuno-suppressive and pro-angiogenic signals and induce the formation of pre-metastatic niches by hematopoietic cells that may shape miRNA profiles in blood cells. Because of these indirect effects of tumors on immune and other circulating cells, the cellular fraction of human peripheral blood might be an appropriate source for biomarker discovery, even if miRNAs released from cancer cells become detectable in plasma or serum when a significant tumor mass has been accumulated [25].

The heat map of the 49 deregulated miRNAs showed an imperfect clustering of the samples. While most of the cancer-free samples clustered separately, some were dispersed within the group of the malignant samples. The separation may be due to a specific expression of miRNAs in benign diseases commonly present in AEC as well as MMP, e.g., asbestosis or pleural plaques. Thus, a detailed characterization of miRNA expression profiles in benign (asbestos-associated) diseases is needed for better differentiation between cases and controls. Furthermore, it is possible that the miRNA expression pattern of AEC clustered between MMP samples arises from early stages of MM when clinical symptoms are still absent. In this case, the miRNA expression profile could indicate an early diagnosis of cancer. However, a follow-up of the AEC is needed to verify this assumption. In this study, the majority of the deregulated miRNAs in MM are down-regulated. This is in accordance with a global down-regulation of miRNAs commonly described in cancer [39], [40] and particularly in MM [15], [17]. However, only miR-103 shows a significant down-regulation in MM.

The identified miR-103 is part of the miR-15/107 group commonly expressed in mammalian tissues [41]. The biological functions of the miR-15/107 group comprise cell division, cellular metabolism, stress response, and angiogenesis, suggesting that the deregulation of these miRNAs contribute to human diseases. In fact, altered expression levels of the miR-15/107 group have been observed in several human cancers [41]. For example, miR-103 and miR-107 are described as possible prognostic markers in esophageal carcinoma [42]. To our knowledge, the present study is the first to show a significant deregulation of miR-103 in MM, including the two histological subtypes epithelioid and biphasic mesothelioma.

Although previous works have shown miR-103 to be down-regulated in several cancers [43], no previous investigation analyzing miRNA expression in MM reported a deregulation of miR-103 miRNA expression [13]–[18]. Such differences in miRNA expression are probably caused by different study designs, mostly regarding the selection of sample types. In this study, the cellular fraction of peripheral blood was used; whereas others examined formalin-fixed paraffin-embedded (FFPE) tissues [16], fresh tissues [14], [15], [17], or cell cultures [13], [18]. The variation in results may be influenced by the size of the study collectives, which ranged between seven [16] and 100 MM cases [18]. Furthermore, the use of different controls, asbestos-exposed individuals in this study and lung cancer patients [17], healthy individuals [18], or commercially available pericardial RNA [14] in others may also be a contributing factor. Additionally, in most studies microarrays were used, which are prone to inconsistent results, mainly due to the different microarray platforms [44].

Subgroup comparisons were performed to evaluate the influence of the potential confounders gender, smoking status, and age. Recently, distinct gender-specific expression patterns for several miRNAs in male and female subjects were published [45]. In our study no gender-specific expression of miR-103 could be observed. This is in accordance with Duttagupta et al., showing no altered miR-103 expression between males and females [45]. A significant lower expression of miR-103 and other miRNAs was described in older individuals [46]. Our study indicated also altered miR-103 levels, but the effect was marginal. Thus, the age of patients should always be taken into account and control groups should be age-matched. The smoking status shows impact on several biological mediators of inflammation [47] and thus, may also influence the miRNA profile. In our study miR-103 is not influenced by the smoking status. This is in accordance with Guled et al., showing several smoking-related miRNAs in a mesothelioma patients, but not miR-103 [14]. However, the assigned subgroups were small, e.g., only 11 subjects were smokers. Thus, the validity of the subgroup analyses is limited and the obtained results may give only hints. A more detailed confounder analysis in larger collectives considering several potential influencing factors as published recently for SMRP, CA 125, and CYFRA 21-1 [5] is needed to evaluate the real impact of potential confounders.

To discriminate MMP from AEC and CGP, respectively, a proper cut-off for miR-103 was determined utilizing ROC analysis. The selected cut-off of 0.621 resulted in 83% sensitivity and 71% specificity for MMP vs. AEC, and in 78% sensitivity and 84% specificity for MMP vs. CGP. The results imply that miR-103 is a potential biomarker of MM. In comparison to miR-126 (73% sensitivity and 74% specificity) [15], miR-103 shows a comparable specificity but slightly higher sensitivity. However, miR-126 has been evaluated in a larger study group (44 mesothelioma patients, 196 asbestos-exposed controls, and 50 healthy controls) [15]. Therefore, the feasibility of miR-103 for detecting MM should also be validated in larger collectives in order to obtain more reliable values for sensitivity and specificity.

As a marker panel is more significant than a single marker for the diagnosis of MM [7], [48], it may be useful to evaluate miR-103 in combination with other biomarkers like SMRP [49] or calretinin [36] to improve sensitivity and specificity. This was already performed for miR-126 and SMRP, suggesting a potential diagnostic biomarker combination for patients with early stages of MM [15]. Nevertheless, to validate the performance of biomarkers for the early detection of cancer, the most suitable design is a prospective study [36].

In conclusion, in this pilot study we show the feasibility of the cellular fraction of peripheral human blood for biomarker discovery, suggesting a promising alternative to plasma or serum. We evaluated miR-103 as a new potential biomarker for the diagnosis of mesothelioma, showing a promising sensitivity and specificity. The suitability of miR-103 alone and in combination with other biomarkers for early detection of mesothelioma needs to be further validated in a prospective study.
